# Silencing of the Prophenoloxidase Gene *BtPPO1* Increased the Ability of Acquisition and Retention of Tomato chlorosis virus by *Bemisia tabaci*

**DOI:** 10.3390/ijms23126541

**Published:** 2022-06-11

**Authors:** Nan Yang, Tianbo Ding, Dong Chu

**Affiliations:** Key Lab of Integrated Crop Pest Management of Shandong Province, College of Plant Health and Medicine, Qingdao Agricultural University, Qingdao 266109, China; qauyangnan@163.com (N.Y.); tianboding@126.com (T.D.)

**Keywords:** *Bemisia tabaci*, Tomato chlorosis virus, prophenoloxidase, RNA interference, virus acquisition, virus retention

## Abstract

Tomato chlorosis virus (ToCV) has seriously impacted tomato production around the world. ToCV is semi-persistently transmitted by the whitefly, *Bemisia tabaci*, which is a serious agricultural pest in the world. However, the interaction mechanism between ToCV and its whitefly vector is still poorly understood. Our previous transcriptome analysis demonstrated that the expression level of an immune-related gene, prophenoloxidase (PPO), in *B. tabaci* increased after ToCV acquisition, which indicates that the PPO may be involved in the interaction mechanism between the ToCV and its vector. To determine the role of the PPO in the acquisition and retention of ToCV by *B. tabaci*, we cloned the complete Open Reading Frames (ORF) of the *BtPPO*s (*BtPPO1* and *BtPPO2*), and then structure and phylogenetic analyses were performed. *BtPPO*s were closely related to the PPO genes of Hemiptera insects. Spatial-temporal expression detection was qualified by using reverse transcription quantitative PCR (RT-qPCR), and this revealed that *BtPPO*s were expressed in all tissues and developmental stages. We found that only *BtPPO1* was significantly upregulated after *B. tabaci* acquired ToCV for 12 and 24 h. According to the paraffin-fluorescence probe-fluorescence in situ hybridization (FISH) experiment, we verified that ToCV and *BtPPO1* were co-located in the thorax of *B. tabaci*, which further revealed the location of their interaction. Finally, the effects of the *BtPPO*s on ToCV acquisition and retention by *B. tabaci* were determined using RNA interference (RNAi). The results showed that the RNAi of the responsive gene (*BtPPO1*) significantly increased the titer of ToCV in *B. tabaci*. These results demonstrate that *BtPPO1* participates in ToCV acquisition and retention by *B. tabaci*.

## 1. Introduction

The sweet potato whitefly, *Bemisia tabaci* (Gennadius) (Hemiptera: Aleyrodidae), is a destructive agricultural pest worldwide. It poses a threat to crop production through direct feeding and honeydew secretion [1,2,3,4]. *B. tabaci* can also transmit more than 300 plant viruses and lead to virus outbreaks [5,6,7]. Tomato chlorosis virus (ToCV) (Closteroviridae: *Crinivirus*) has spread from north central Florida (USA) into many countries, including China, accompanied by the outbreak of *B. tabaci* [8,9,10]. ToCV can infect numerous plants, and the spread of this virus depends on *B. tabaci* in many places [11,12,13,14]. In recent years, ToCV was mainly transmitted by *B. tabaci* MED (formerly biotype Q, herein called *B. tabaci*), and its incidence was enhanced with the increasing *B. tabaci* populations [9], which show that the epidemiology of ToCV in the field is closely associated with this vector whitefly. However, little is known about the mechanism of interaction between ToCV and *B. tabaci*.

The mechanism of plant virus transmission by insects is complex and involves interactions among plants, insects, and plant viruses [15]. Many genes in vector insects can be involved in the transmission of plant viruses [16,17,18,19]. The expression level of the innate immune gene, prophenoloxidase (PPO), in *B. tabaci* was upregulated after ToCV acquisition by *B. tabaci* [20], which suggested that PPO may play an important role in the resistance of ToCV by *B. tabaci*. Insect PPO is a type 3 copper-containing protein, which is involved in defense against bacteria, fungi, and viruses [21]. Similarly, the expression level of PPO increased in *Frankliniella occidentalis* after acquisition of tomato spotted wilt virus (TSWV) [22]. Thus, we believe that *BtPPO*s may play a role in ToCV acquisition and retention by *B. tabaci*, which needs to be verified.

To study the role of the *BtPPO*s in *B. tabaci*, we cloned the complete ORF of *BtPPO*s in *B. tabaci* and examined its expression characteristics in different stages and tissues. We qualified the changes of the expression of these PPO genes after ToCV acquisition using RT-qPCR. We then determined the location of the ToCV and *BtPPO1* by fluorescence in situ hybridization (FISH). Finally, we used the RNA interference (RNAi) method to reveal the effects of *BtPPO*s on ToCV acquisition and retention by *B. tabaci.*

## 2. Results

### 2.1. Analysis of Complete Sequences of BtPPOs in Bemisia tabaci

**Figure 1:**
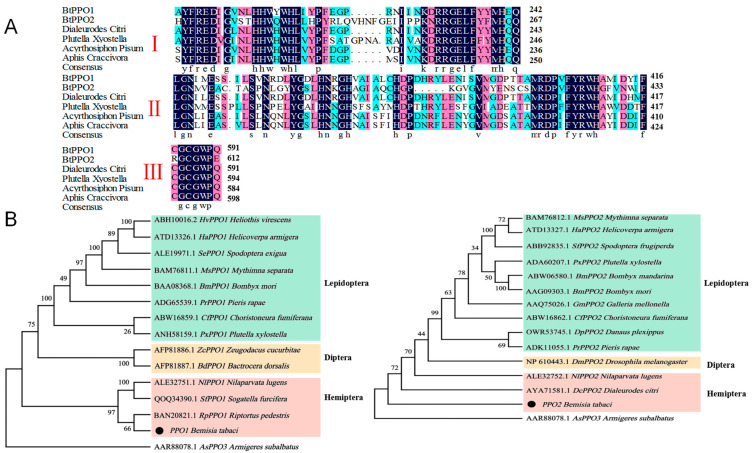
Comparison of prophenoloxidase genes’ sequences and phylogenetic analysis. (**A**) Alignments of the deduced amino acid sequences of prophenoloxidase genes in *Bemisia tabaci* with other insects. Hyphens (-) are used to indicate regions where gaps have been introduced to maximize homology. (**B**) Phylogenetic trees of prophenoloxidase genes with other insect prophenoloxidases. The black dot represents the deduced amino acid sequences of prophenoloxidase genes in *Bemisia tabaci*. The cladogram was constructed by MEGA software using the neighbor-joining method. *Armigeres subalbatus* prophenoloxidase-3 gene was used as an outgroup (*Dialeurodes citri*: AYA71581.1; *Plutella xyostella*: ACS36209.1; *Acyrthosiphon pisum*: XP_001949307.1; *Aphis craccivora*: KAF0771674.1). Region I: copper binding site A (Cu^A^), region II: copper binding site B (Cu^B^), and region III: thiol ester region-like motif. Note: identical amino acids (equal to 100%) are framed by the filled dark blue boxes, identical amino acids (greater than or equal to 75%) are framed by the filled pink boxes, and identical amino acids (greater than or equal to 50%) are framed by the filled light blue boxes.

Using RT-PCR, the complete ORF of the two PPO genes were cloned and sequenced. The ORF lengths of the *BtPPO1* and *BtPPO2* were 2148 and 2151 bp (Figure S1), respectively. The predicted molecular weights of the BtPPOs were 82.3 (BtPPO1) and 83 kDa (BtPPO2), and the predicted PIs were 6.44 (BtPPO1) and 6.52 (BtPPO2). The BtPPOs contained three categories of conserved domains (Figure 1A). The phylogenetic trees indicated two PPO genes from one cluster with other PPO subfamily genes. Within the Hemiptera clade, BtPPO1 was first grouped with PPO1 of *Riptortus pedestris*, with a 71% bootstrap value. Interestingly, the phylogenetic relationships of BtPPO2 were closely related to the *Dialeurodes citri* (Figure 1B).

### 2.2. Spatiotemporal Expression Profiles of BtPPOs in Bemisia tabaci

**Figure 2:**
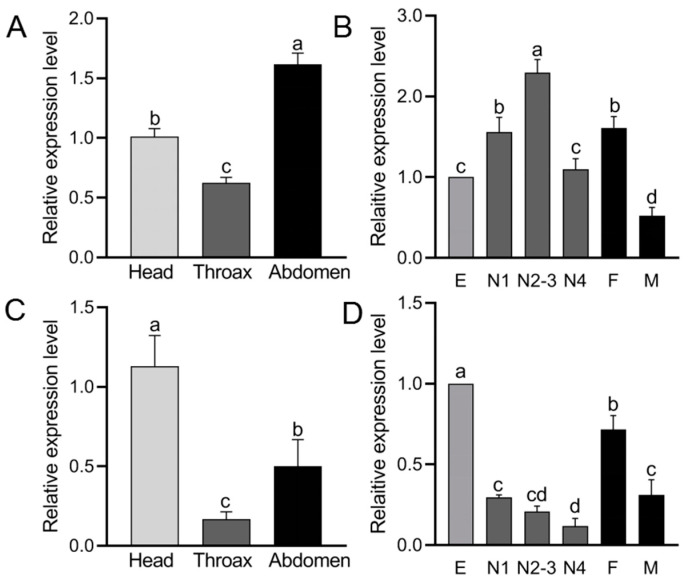
Expression profiles of the prophenoloxidase genes in different tissues and stages of *Bemisia tabaci*. (**A**) *BtPPO1* expressions in different tissues. (**B**) *BtPPO1* expressions in different stages. (**C**) *BtPPO2* expressions in different tissues. (**D**) *BtPPO2* expressions in different stages. Relative expression levels of the prophenoloxidase genes in eggs (E), first stage nymphs (N1), second to third stage nymphs (N2–3), fourth stage nymphs (N4), female adults (F), and male adults (M). Data of different stages and tissues are presented as the mean ± SE for three and four independent replicates, respectively. The data were normalized to the expression of SDHA and EF-1α. The bars with different small letters in the figures are significantly different according to the one-way ANOVA, followed by Tukey’s multiple comparison test (*p* < 0.05).

The RT-qPCR results showed that *BtPPO1* had the highest expression in the abdomen (Figure 2A), and in the developmental stages, the expression of *BtPPO1* increased from egg to third instar nymph. Expressions of *BtPPO1* were highest in the second to third nymphs (Figure 2B), while the *BtPPO2* had the highest expression in the head. Both of the genes had the lowest expression levels in the thorax (Figure 2C). Interestingly, the *BtPPO2* had the highest expression in egg and decreased from the first nymphs to the fourth nymphs (Figure 2D). The same phenomenon was seen, where both *BtPPO1* and *BtPPO2* had higher expression in female adults than in male adults.

### 2.3. Expression Profiles of BtPPOs in Bemisia tabaci after ToCV Acquisition

**Figure 3:**
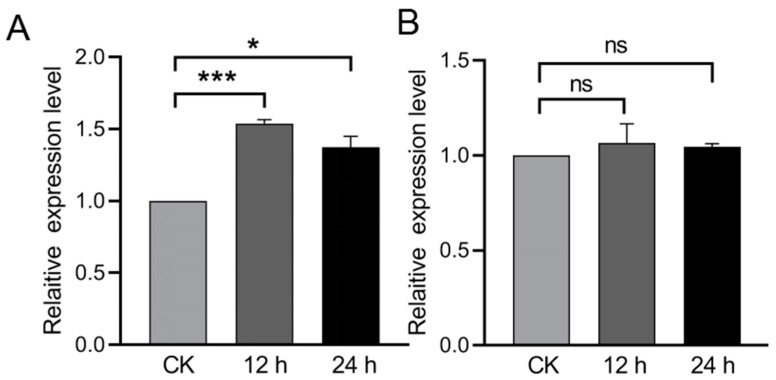
Expression profiles of prophenoloxidase genes in *Bemisia tabaci* after feeding on ToCV-infected tomatoes for different durations. (**A**) *BtPPO1* and (**B**) *BtPPO2*. Data are presented as the mean ± SE for four independent replicates. The data were normalized to the expression of SDHA and EF-1α. Asterisks indicate significant differences between the treatment and corresponding untreated control according to the Student’s *t*-test. * *p* < 0.05, *** *p* < 0.001, ns indicates no significant difference.

Compared with *B. tabaci* feeding on healthy tomato plants, the expression of *BtPPO1* increased significantly by 1.53-(*p* < 0.001) and 1.37-fold (*p* < 0.05) after ToCV acquisition at 12 and 24 h, respectively (Figure 3A). However, *BtPPO2* had no significant change after feeding on ToCV-infected tomatoes (Figure 3B).

### 2.4. Distribution of BtPPO1 and ToCV in Bemisia tabaci

**Figure 4:**
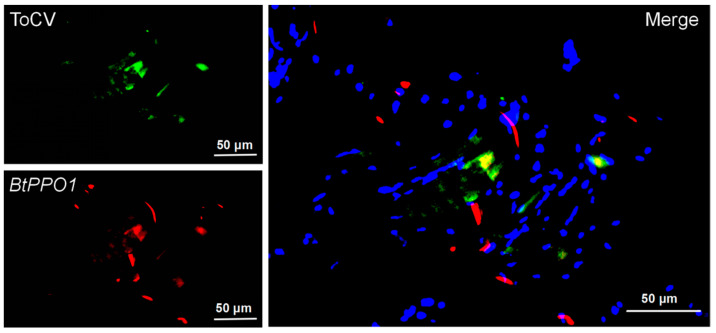
Fluorescence in situ hybridization analysis of symbionts *BtPPO1* and ToCV in *Bemisia tabaci* adult thorax. ToCV glows green by excitation wavelength 465–495 nm and emission wavelength 515–555 nm, and BtPPO1 glows red by excitation wavelength 510–560 nm and emission wavelength 590 nm. The nuclear staining by DAPI was blue under ultraviolet excitation.

The *BtPPO1* and coat protein of ToCV were located in the thorax of viruliferous whitefly, and the positions of green fluorescence and red fluorescence were highly coincident in the thorax of *B. tabaci* (Figure 4).

### 2.5. Efficiency of RNAi and Its Effects on ToCV Acquisition by Bemisia tabaci

**Figure 5:**
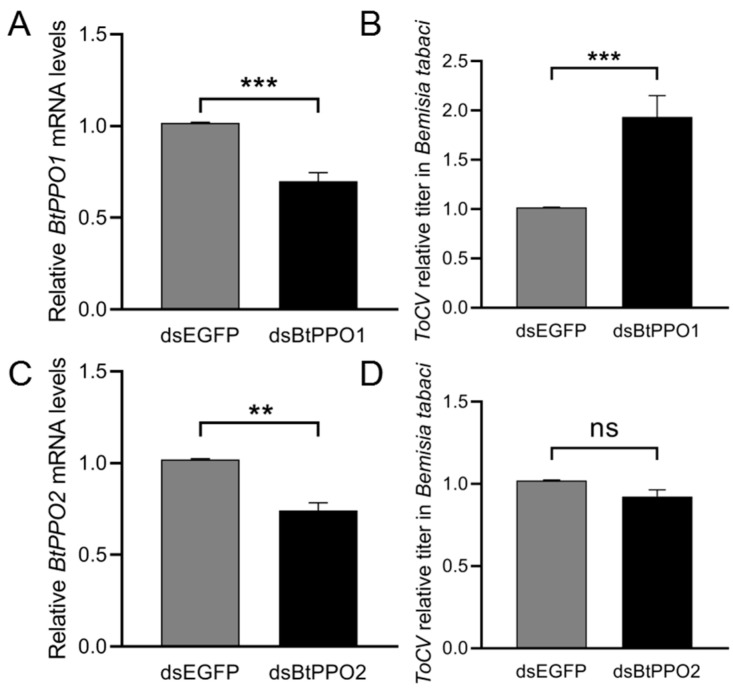
Efficiency of RNA interference and the effects on ToCV acquisition by *Bemisia tabaci*. (**A**) Relative mRNA levels of *BtPPO1* in *B*. *tabaci* adults after feeding on a diet containing dsEGFP and dsBtPPO1. (**B**) Effect of *BtPPO1* gene silencing on ToCV titer in *B*. *tabaci* feeding on ToCV-infected tomato plants at 24 h. Data of (**A**,**B**) presented as the mean ± SE for eight independent replicates. (**C**) Relative mRNA levels of *BtPPO2* in *B*. *tabaci* adults after feeding on a diet containing dsEGFP and dsBtPPO1. (**D**) Effect of *BtPPO2* gene silencing on ToCV titer in *B*. *tabaci* feeding on ToCV-infected tomato plants at 24 h. Data of (**C**,**D**) presented as the mean ± SE for six independent replicates. The data were normalized to the expression of SDHA and EF-1α. Asterisks’ indicate significant differences between the treatment and the corresponding untreated control according to the Student’s *t*-test. ** *p* < 0.01, *** *p* < 0.001, ns indicates no significant difference.

After ingestion of dsBtPPO1 for 72 h, the relative expression level of mRNA *BtPPO1* in *B. tabaci* adults significantly decreased (*p* < 0.001) (expression decreased by 30%) compared with the level in adults after feeding on a diet containing dsEGFP (Figure 5A). Likewise, the same result appeared in the dsBtPPO2 treatment (expression decreased by 26%, *p* < 0.01) (Figure 5C). The virus titer in *B. tabaci* treated with dsBtPPO1 was significantly (*p* < 0.001) higher than the titer in the control treated with dsEGFP (Figure 5B). However, the virus titer in *B. tabaci* had no discrepancies between dsBtPPO2 treatment and dsEGFP treatment (Figure 5D).

### 2.6. Efficiency of RNAi and Its Effects on ToCV Retention by Bemisia tabaci

**Figure 6:**
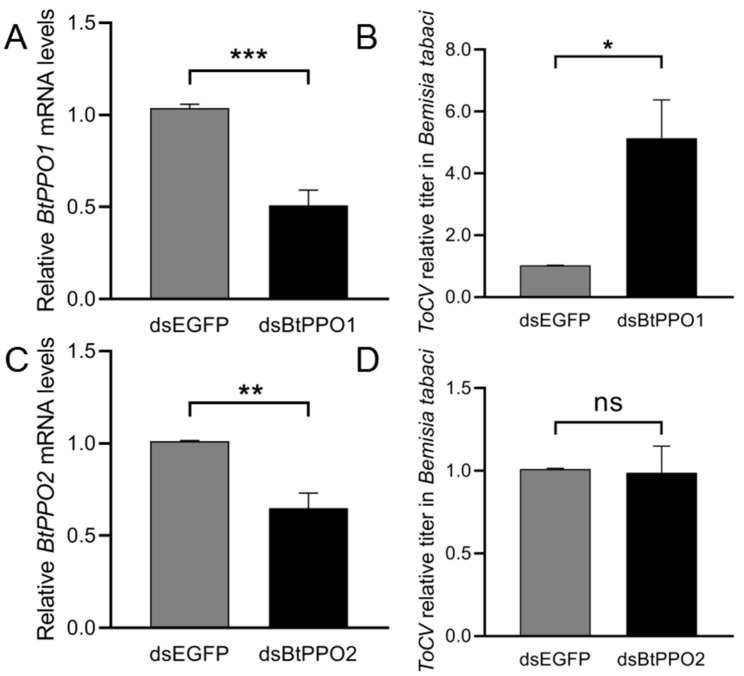
Efficiency of RNA interference and ToCV titer in *Bemisia tabaci* after RNA interference. (**A**) Relative mRNA levels of BtPPO1 in *B. tabaci* adults after feeding on a diet containing dsEGFP and dsBtPPO1. (**B**) Effect of BtPPO1 gene silencing on ToCV titer in *B. tabaci*. Data of (**A**,**B**) presented as the mean ± SE for nine independent replicates. (**C**) Relative mRNA levels of BtPPO2 in *B. tabaci* adults after feeding on a diet containing dsEGFP and dsBtPPO2. (**D**) Effect of BtPPO2 gene silencing on ToCV titer in *B. tabaci*. Data of (**C**,**D**) presented as the mean ± SE for six independent replicates. The data were normalized to the expression of SDHA and EF-1α. Asterisks’ indicate significant differences between the treatment and the corresponding untreated control according to the Student’s *t*-test. * *p* < 0.05, ** *p* < 0.01, *** *p* < 0.001, ns indicates no significant difference.

After ingestion of dsBtPPOs for 72 h, the relative expression level of mRNA *BtPPO*s in *B. tabaci* adults significantly decreased (expression decreased by BtPPO1: 50%, *p* < 0.001 and BtPPO2: 35%, *p* < 0.01) compared with the level in adults after feeding on a diet containing dsEGFP (Figure 6A,C). The virus titer in *B. tabaci* treated with dsBtPPO1 was significantly (*p* < 0.05) higher than the titer in the control treated with dsEGFP (Figure 6B). On the contrary, the virus titer in *B. tabaci* treated with dsBtPPO2 had no differences comparing to the dsEGFP-treated group (Figure 6D).

## 3. Discussion

Many genes in the vector insects are involved in plant virus transmission. Meanwhile, many immune-related genes in vector insects are involved in the transmission of plant viruses [18,23]. For example, the expression of the immune gene cathepsin B was upregulated in *Sitobion avenae* (Fabricius) exposed to *Barley yellow dwarf virus* (BYDV) [20]. Similarly, the expression of the antiviral immune genes hemocyanin and cathepsin in *B. tabaci* can be induced by *Tomato yellow leaf curl virus* (TYLCV) [24]. ToCV has caused severe damage to plants in many regions [10,14]. However, few studies have focused on the interaction between ToCV and its vector, *B. tabaci* [25,26]. The expression level of PPO in *B. tabaci* was upregulated after ToCV acquisition [21]. In insects, PPO is an important innate immunity protein that induces melanization after activation and induces cellular and humoral immunity. It is essential for preventing the invasion of pathogenic microorganisms [27,28,29]. For example, PPO2 in *Drosophila melanogaster* increases survival after infection with Gram-positive bacteria and fungi and promotes melanization in insect host defense [30,31,32,33,34]. However, the potential role of the *BtPPO*s in *B. tabaci* during ToCV acquisition and retention remains poorly understood.

We cloned the complete ORF of the immune genes, *BtPPO*s, in *B. tabaci*. Alignments of the deduced amino acid sequences of *BtPPO*s with other insects from Hemiptera showed that the BtPPOs have three domains, namely, Cu^A^, Cu^B^, and a thiol ester region-like motif [35], which was common to all invertebrate PPOs. According to the phylogenetic analysis, the BtPPOs were grouped together with Hemiptera. The degree of sequence identity within an order was generally greater than among orders. The relatively high sequence similarity between *B. tabaci* and other Hemiptera insects indicates that *BtPPO*s may perform a vital function similar to other insects.

Our results identified two PPO genes in *B. tabaci* that have significantly different expressions in various development stages and tissues. *BtPPO1* has the highest expression in the abdomen, while *BtPPO2* has the highest expression in the head. The expression of *BtPPO1* fluctuated during all life stages of *B. tabaci*, and had the lowest expression in eggs, which suggests that *BtPPO1* has the most important role in the life stages from larva to adult, and the second- to third-stage nymphs had greater amounts than the other developmental stages. Interestingly, the expression of a PPO, *PXPPO2*, in *Plutella xylostella* was also higher in the third instar larvae than in other stages [35]. These results indicate that *BtPPO1* may play an important role during larval development.

However, we found that the expression level of only one of the *BtPPO*s, *BtPPO1*, could respond to ToCV acquisition (Figure 3A), which confirmed the transcriptome analysis results of Ding et al. [21]. On the other hand, the *BtPPO2* had no changes after acquisition of ToCV at different times, which indicated that *BtPPO2* may not be involved in the interaction between ToCV and *B. tabaci*. PPO2 had a negative effect on the activity of PPO1 to a certain extent. PPO1 and PPO2 in Drosophila have different blackening patterns, which explains the reasons why the two PPO genes play different roles in immune response. Their different roles may be caused by the different localization of PPO1 and PPO2 in hemolymph and lens cells, respectively [33]. At the same time, the results of fluorescence in situ hybridization showed that *BtPPO1* and ToCV were concentrated in the thorax of *B. tabaci,* which suggested that *BtPPO1* and ToCV may interact in the thorax.

The RNAi of *BtPPO1* significantly changed the acquisition and retention of ToCV by *B. tabaci*. In contrast, the *BtPPO2* had no effects in the acquisition and retention of ToCV by *B. tabaci.* This showed that the decrease of *BtPPO1* expression can improve the ability of ToCV acquisition and retention by *B. tabaci*. *BtPPO1* may not be conducive to ToCV survival in *B. tabaci*. The FISH results showed that *BtPPO1* and ToCV were located in the thorax of *B. tabaci*, which is consistent with the finding that semi-persistent virus particles may exist in the insect foregut [36]. The same results appeared in the semi-persistent virus *lettuce infectious yellows virus* (LIYV), where LIYV virions were found to be retained in the foreguts of new world (NW) species of *Bemisia tabaci* [37]. However, in addition to blood cells, PPO is also distributed in the foregut and hindgut of some insects [38]. This finding showed that the immune-related PPO genes in the foregut of *B. tabaci* may be involved in the interaction between plant virus and vector insects.

We determined the function of *BtPPO1* in the interaction between *B. tabaci* and ToCV, but the connection between the PPO cascade and signaling pathway requires further study. For example, there is a relationship between the *PPO* cascade and the Toll signaling pathway [39], and upregulated genes in the Toll signaling pathway can influence the resistance to virus infection [40]. Hence, *BtPPO1* may participate in the Toll signaling pathway to control ToCV acquisition and retention by *B. tabaci*.

In summary, we identified and characterized the *BtPPO1* gene in *B. tabaci* and confirmed that the *BtPPO1* gene participates in ToCV acquisition and retention by *B. tabaci*. The results indicated that *BtPPO1* was not conducive to acquire and retain ToCV, which provides new insight into the interaction between the plant virus and the vector whitefly. However, the interaction between PPO and *B. tabaci* should be further studied to reveal the mechanism of the acquisition and retention of ToCV by *B. tabaci*.

## 4. Materials and Methods

### 4.1. Insects and Plants

*B. tabaci* used in this study were initially collected in a field in Shandong Province in 2017 and maintained on cotton plants: at 27 ± 1 °C, 60% ± 5% relative humidity (RH), and a 16:8 h (L:D) photoperiod. The *B. tabaci* adults were identified as a MED species by using the mitochondrial cytochrome oxidase I genes (mtCOI) PCR-RFLP method [41]. The cotton plants (non-host plant of ToCV), *Gossypium hirsutum* L. (Lu-Mian 28, Shandong Cotton Research Center, Jinan, China), and the tomato plants (host plant of ToCV), *Solanum lycopersicum* L. (Zhong-za 9, Chinese Vegatable Industry Technology Co., Ltd., Beijing, China), were cultivated using the formula soil in a greenhouse under the conditions: 27 ± 1 °C, 60% ± 5% RH, and a 16:8 h (L:D) photoperiod.

### 4.2. Full-Length cDNA Cloning

About 30 *B. tabaci* adults were used to extract total RNA with TRIzol reagent following the manufacturer’s instructions (Thermo Fisher, Waltham, MA, USA). The purity and concentration of the RNA were determined using a NanoDrop spectrophotometer (IMPLEN GmbH, Los Angeles, CA, USA). A 1 μg quantity of the total RNA was reverse transcribed to produce cDNA using the PrimeScript RT Reagent Kit with gDNA Eraser (TaKaRa, Dalian, China). Based on the genome [42], gene-specific primers (Table S1) were designed to obtain the complete ORF. The PCR reaction was conducted according to the manufacturer of Premix Taq™ (TaKaRa Taq™ Version 2.0) (TaKaRa, Dalian, China). According to the genes’ expected sizes, we used 1% agarose gel electrophoresis to separate the target genes (*BtPPO1* and *BtPPO2*). The genes were purified using a SteadyPure Agarose Gel DNA Purification Kit AG21005 (Accurate Biotechnology Co., Ltd., Changsha, China) and then cloned into the pMD-18T vector (TaKaRa, Dalian, China).

### 4.3. Sequence Analysis of BtPPOs in Bemisia Tabaci

The ExPASy translation tool [43] (http://web.expasy.org/translate/ (accessed on 5 May 2022)) was used to deduce the protein sequence, and the isoelectric point (pI) and molecular weight (MW) were predicted by the ExPASy proteomics tool Compute pI/MW [44]. A phylogenetic tree was generated by ClustalW [45] alignment of the full-length amino acid sequences of *BtPPO1* with other PPO subfamily genes using the neighbor-joining method in MEGA X (Auckland, New Zealand) with 1000 bootstrap replications. Amino acid alignment with other sequences was conducted with the DNAMAN v.6.03 program (San Ramon, CA, USA).

### 4.4. Expression of BtPPOs in Different Tissues and Developmental Stages

To investigate the expression of the *BtPPO*s in different tissues and developmental stages, we collected four replicates of tissues from the head, thorax, and abdomen. Three replicates of female adults at the following six stages: eggs (1000), first-stage nymphs (500), second- to third-stage nymphs (500) (the second and third stages are difficult to distinguish), fourth-stage nymphs (300), 50 adult females and adult males, and 100 of each tissue as a biological replicates, were used for each sample, and the expression was quantified using RT-qPCR.

### 4.5. Expression of BtPPOs in Bemisia tabaci after ToCV Acquisition

To understand the expression of the *BtPPO*s after the acquisition of ToCV, the *B. tabaci* adults were fed on ToCV-infected tomato leaves for 12 and 24 h, respectively. These ToCV-infected tomato plants were used as viral sources 4 weeks post-inoculation with the virus. After *B. tabaci* acquired the virus, we detected the relative *BtPPO*s mRNA level changes by RT-qPCR. Non-virus feeding was used as a control (CK), and virus feeding for 12 and 24 h were used as treatment. Four replicates (*n* = 30 adults per replicate) were performed for each treatment.

### 4.6. Paraffin-Double-Fluorescence Probe-Fluorescence In Situ Hybridization

*B. tabaci* adults (acquired ToCV for 48 h) were immediately placed into the fixed fluid after 12 h. The *B. tabaci* was dehydrated by gradient alcohol and paraffin-embedded. The paraffin was sliced, and slices were dewaxed and dehydrated. Proteinase K (20 μg/mL) working solution (Servicebio, Wuhan, China) was added to cover objectives, incubated at 37 °C for 15 min, then washed three times (5 min each) in PBS (pH 7.4) (Servicebio, Wuhan, China). After washing in PBS for 15 min, the slices were rinsed in hybridization buffer (20 mM Tris-HCl, pH 8.0, 0.9 M NaCl, 0.01% (wt./vol) sodium dodecyl sulfate, 30% (vol/vol) formamide) (Servicebio, Wuhan, China) for the pre-hybrid (without the probe). Then, 1 μmol of the fluorescent BtPPO1 probe (conjugated with Cy3) was added into the slices. The slices were rinsed in hybridization buffer again. After that, 1 μmol of the fluorescent ToCV probe (conjugated with FAM) was added into the slices. Then, the hybridized slices were rinsed three times in hybridization buffer and incubated with DAPI (Servicebio, Wuhan, China) for 8 min in the dark, then mounted before taking photos with a positive fluorescence microscope (Nikon, Tokyo, Japan). Probe sequences are listed in Table S1.

### 4.7. Reverse Transcription Quantitative PCR (RT-qPCR)

The relative transcription levels of the PPO mRNA were examined by RT-qPCR. The cDNA synthesis was performed as described above. The expression stabilities of five genes, namely, β-Actin, elongation factor 1 alpha (EF-1α), nicotinamide adenine dehydrogenase (NADH), heat shock protein 90 (HSP90), and succinate dehydrogenase complex subunit A (SDHA), were also evaluated. We selected EF-1α and SDHA as endogenous reference genes for the expression of *BtPPO*s in samples that received different treatments (Figure S2). The reactions were performed in a 20 μL mixture containing 2 μL of cDNA, 10 μL of TB Green^®^ Premix Ex Taq™ (Tli RNaseH Plus) (TaKaRa, Dalian, China), 0.8 μL of each primer, and 6.4 μL of DEPC-treated water. The optimized real-time PCR program consisted of an initial step at 95 °C for 30 s, followed by 40 cycles of 95 °C for 5 s, and 60 °C for 30 s. After the cycling protocol, the double-stranded DNA was denatured by increasing the temperature from 60 to 95 °C (0.6 °C s^−1^) to obtain the melting curves. The RT-qPCR amplifications were carried out in 96-well plates (NEST, Wuxi, China), and the assays were run on a JENA qTOWER 2.2 system (Analytikjena, Jena, Germany). Quantification of the transcript level of the genes was conducted using the 2^−ΔΔCt^ method [46].

### 4.8. Double-Stranded RNA (dsRNA) Synthesis

The fragments of *BtPPO1*, *BtPPO2,* and enhanced green fluorescent protein gene (EGFP) were amplified by reverse transcription PCR (RT-PCR) using specific primers conjugated with 20 bases of the T7 RNA polymerase promoter (Table S1). The PCR products (1 μg) were used as templates for dsRNA synthesis using the TranscriptAid T7 High-Yield Transcription Kit (Thermo Fisher, Waltham, MA, USA). After synthesis, the dsRNA was ethanol-precipitated, re-suspended in DEPC-treated water, and quantified with a NanoDrop spectrophotometer (IMPLEN GmbH, Los Angeles, CA, USA). The concentrations of dsBtPPOs (dsBtPPO1 and dsBtPPO2) and dsEGFP were 800 ng/μL. The other end of the tube was covered with gauze and this side was placed downward. There were two independent experiments for *BtPPO1* and *BtPPO2*, respectively.

### 4.9. Functional Analysis of BtPPOs in Acquisition Ability of Virus by RNAi

Whitefly adults (incubated within 72 h) were collected and placed into a 50 mL tube (2.5 cm in diameter, 6.5 cm in height). Both sides of the tube were open, and one side was separated by a layer of PTFE film that was filled with 2.5 mL of 800 ng/μL of dsBtPPOs (dsBtPPO1 and dsBtPPO2). The control group was fed with an equivalent concentration of dsEGFP. After continuously ingesting dsEGFP- and dsBtPPOs-containing diets for 72 h, the adults were fed on ToCV-infected tomatoes for 24 h. Eight replicates (*n* = 30 adults per replicate) for each treatment, dsEGFP and dsBtPPO1, and six replicates (*n* = 30 adults per replicate) for dsBtPPO2 and dsEGFP, were exposed to ToCV-infected tomato leaves for 24 h and used to extract total RNA to detect the virus titer in *B. tabaci* and determine potential knockdown of targeted genes. The effect of *BtPPO*s on the ability of *B. tabaci* to acquire the virus was verified by the above steps.

### 4.10. Functional Analysis of BtPPOs in Retention Ability of Virus by RNAi

Firstly, whitefly adults (incubated within 72 h) were exposed to ToCV-infected tomato leaves to acquire ToCV for 48 h, and then the adults continuously ingested dsEGFP- and dsBtPPOs-containing diets for 72 h. Nine replicates (*n* = 30 adults per replicate) for each treatment, dsEGFP and dsBtPPO1, and six replicates for dsEGFP and dsBtPPO2, were used to extract total RNA to determine potential knockdown of targeted genes and the virus titer in *B. tabaci.* The effect of *BtPPO*s on the ability of *B. tabaci* to retain the virus was verified by the above steps.

### 4.11. Statistical Analysis

The data are presented as means ± SE, and they were analyzed by using SPSS version 21 (SPSS Inc., Chicago, IL, USA). The statistical significance of the gene expressions in different stages and tissues of *B. tabaci* was calculated using Tukey’s multiple comparison test. Differences between PPO expression in *B. tabaci* after feeding on ToCV-infected tomato leaves for different durations and the silencing efficiency of RNAi and ToCV titer in *B. tabaci* were compared using Student’s *t*-test. All RT-qPCR values are relative to a reference treatment. All Ct values for all RT-qPCR experiments are shown in Table S2.

## 5. Conclusions

In summary, we cloned and identified the *BtPPO1* gene in *B. tabaci* and confirmed that the *BtPPO1* gene participates in ToCV acquisition and retention by *B. tabaci*. The results indicated that *BtPPO1* was not conducive to acquire and retain ToCV, which provides new insight into the interaction between the plant virus and the vector whitefly. We used RNAi to reveal the role of PPO in the transmission of ToCV by *B. tabaci*. *BtPPO1* can inhibit the acquisition and retention ability of ToCV by *B. tabaci*. These results provide a reference for revealing the molecular mechanism of interactions between *B. tabaci* and ToCV.

## Data Availability

Not applicable.

## Supplementary Materials

The following supporting information can be downloaded at: https://www.mdpi.com/article/10.3390/ijms23126541/s1.
