# Supplementary figures and images for: Silencing of the Prophenoloxidase Gene BtPPO1 Increased the Ability of Acquisition and Retention of Tomato chlorosis virus by Bemisia tabaci
Source: Int J Mol Sci. 2022 Jun 11;23(12):6541. doi: 10.3390/ijms23126541 (PMC9223377; doi:10.3390/ijms23126541)

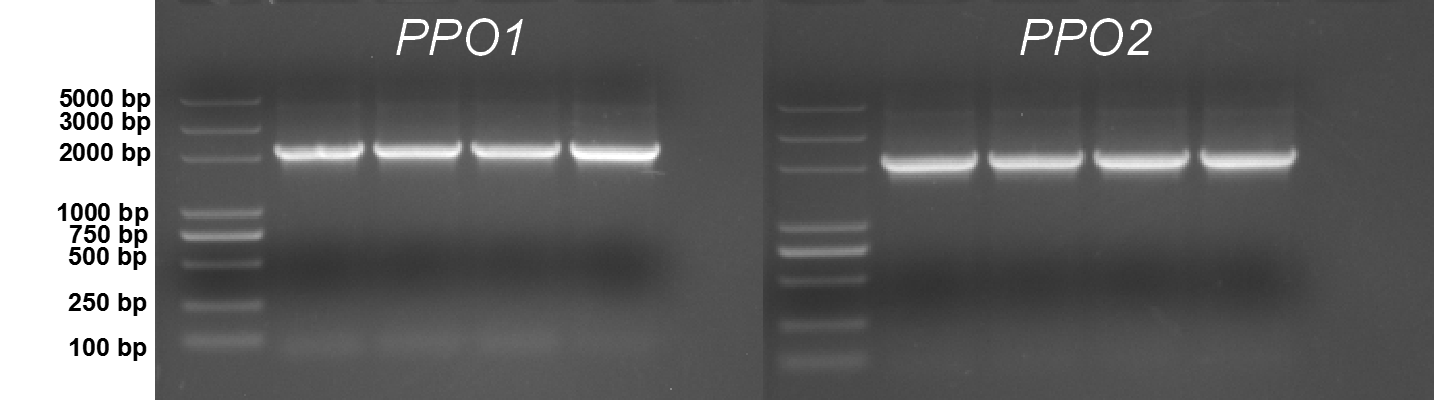

Supplement: Supplementary file 1 [file ijms-23-06541-s001.zip › Figure S1.tif]

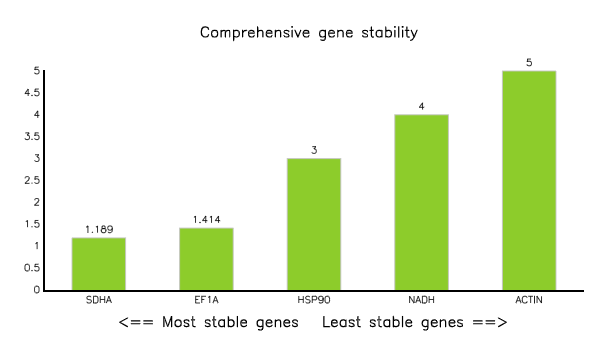

Supplement: Supplementary file 1 [file ijms-23-06541-s001.zip › Figure S2.tif]
